# Pancreatic Sarcoidosis: A Very Rare Presentation of the Disease

**DOI:** 10.7759/cureus.7996

**Published:** 2020-05-06

**Authors:** Hanan Ibrahim, Hazem Zebda, Alireza Meysami

**Affiliations:** 1 Internal Medicine, Henry Ford Hospital, Detroit, USA; 2 Rheumatology, Henry Ford Health System, Detroit, USA

**Keywords:** sarcoidosis, pancreatitis, abdominal pain

## Abstract

Sarcoidosis is a multisystem chronic granulomatous disease of unknown etiology that predominantly affects the lungs, but the disease process can affect any other organ. Gastrointestinal involvement remains rare, thus diagnosis remains challenging. Obtaining laboratory findings and pathological evidence of the non-caseating non-necrotizing granulomas in the appropriate clinical picture can help guide the diagnosis. We present a rare case of the disease, with involvement of the pancreas.

## Introduction

Sarcoidosis is a granulomatous inflammatory disease that affects multiple organs of the body and most likely to affect the lungs. It also affects any other organ in the human body. One of the organs that is rarely involved in the disease is the pancreas. According to literature, about 1%-5% of patients with systemic sarcoidosis have pancreatic involvement upon autopsies [1]. The disease presentation and diagnosis remains challenging; a high level of suspicion with a combination of laboratory and imaging studies needed to reach the final diagnosis. The disease has a good response to steroids, which improves outcomes dramatically [2] . Here we present a case of a rare multisystem sarcoidosis with pancreatic involvement.

## Case presentation

A 41-year-old female patient with a past medical history of iron-deficiency anemia was referred to the rheumatology clinic for evaluation of possible sarcoidosis. 

**Figure 1:**
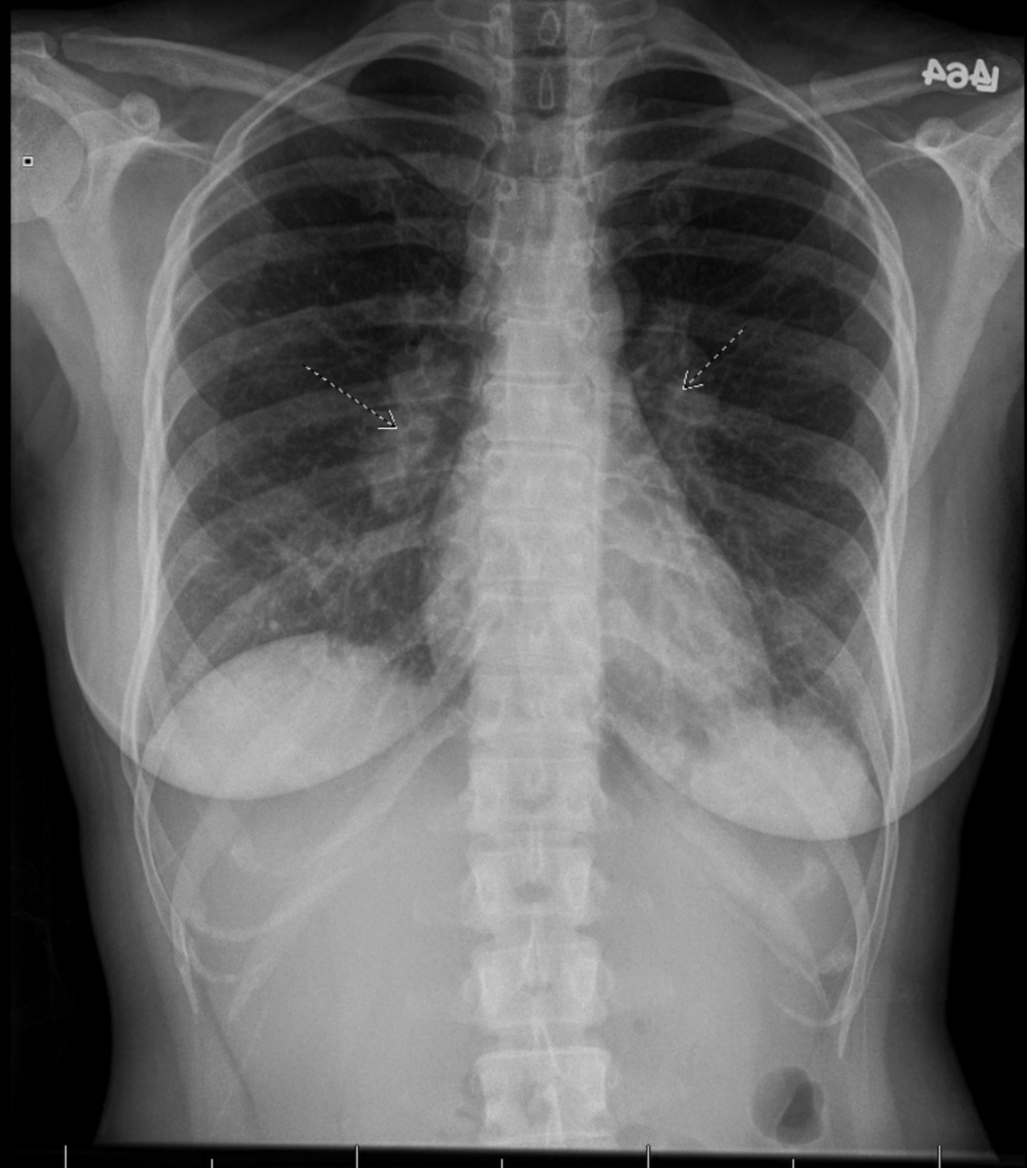
Chest x-ray showing bilateral hilar lymphadenopathy

She initially presented to the gastroenterology clinic after which she had significant epigastric abdominal pain brought on by eating. Her pain was associated with generalized fatigue. She underwent esophagogastroduodenoscopy with sampling of the gastric and duodenal mucosa, which revealed mild chronic inflammation. There were no signs of granulomatous disease in the sample. About a month after her endoscopy, she started having blurry vision in both of her eyes along with pain upon eye movement. She was evaluated by her ophthalmologist and was diagnosed with anterior chamber uveitis with granulomatous keratic precipitates. She was started on prednisolone acetate eye drops, which improved her symptoms significantly. Upon tapering the steroids down, her symptoms began to worsen again. A workup was initiated at that time to rule out other causes. Laboratory workup was significant for an elevated C-reactive protein (1.4 mg/L), elevated angiotensin-converting enzyme (ACE) level to 274 U/L (normal range: 8-52 U/L), and a lysozyme level to 22.4 mcg/mL (normal range 5-11 mcg/mL). Antinuclear antibodies, syphilis serology, toxoplasma serology, tuberculosis, and Borrelia serology were unremarkable. A chest x-ray was done, which showed mediastinal and hilar lymphadenopathy bilaterally (Figure 1).

**Figure 2:**
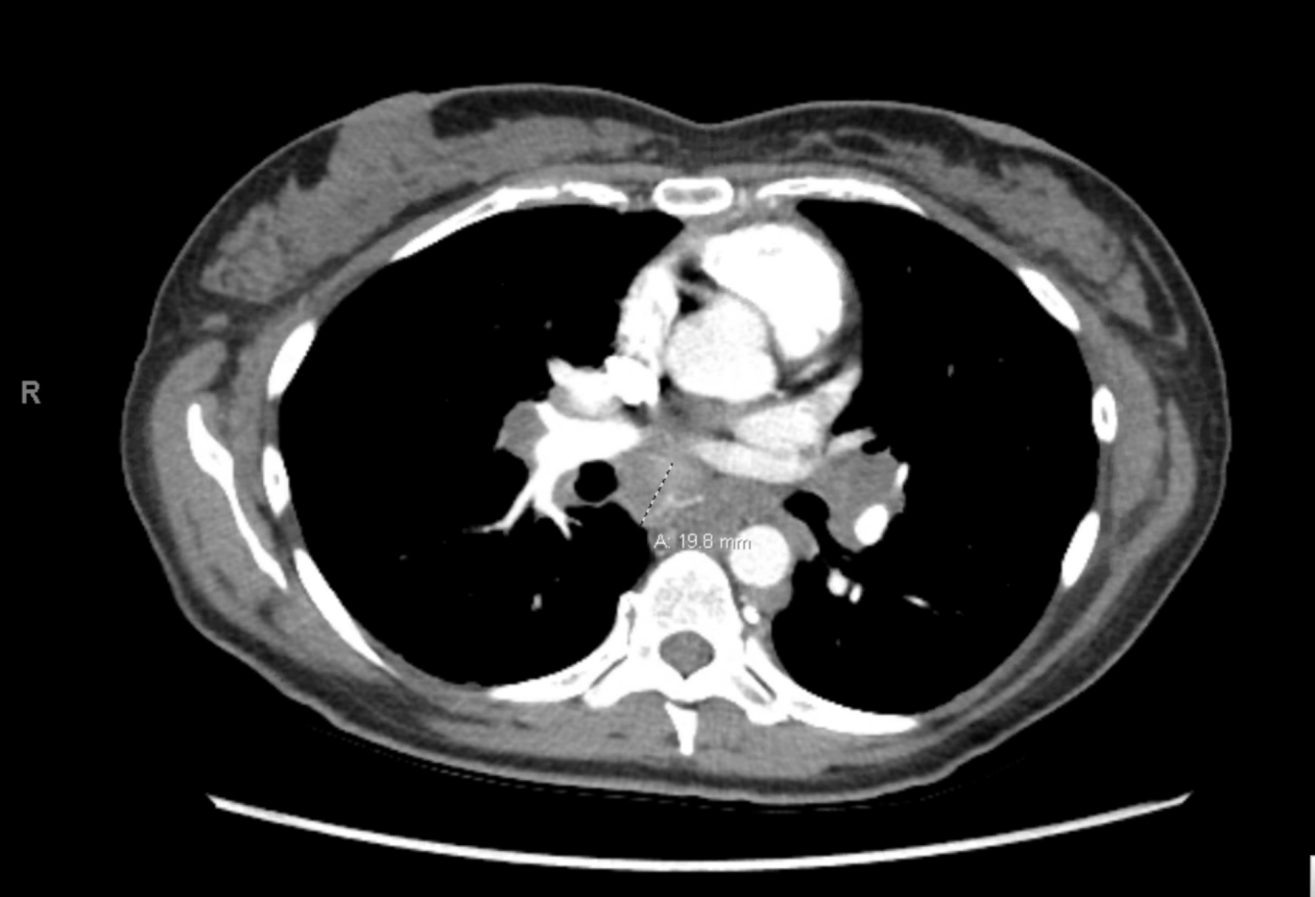
CT of the chest revealing mediastinal and hilar lymphadenopathy suspicious for sarcoidosis

She also noted a bilateral skin rash on her lower extremities; a skin biopsy was taken and showed non-caseating granulomatous dermatitis. Due to previous chest x-ray findings and continuous abdominal pain, she underwent computed tomography of the chest and abdomen, which showed mediastinal and hilar lymphadenopathy suspicious for sarcoidosis (Figure 2).

**Figure 3:**
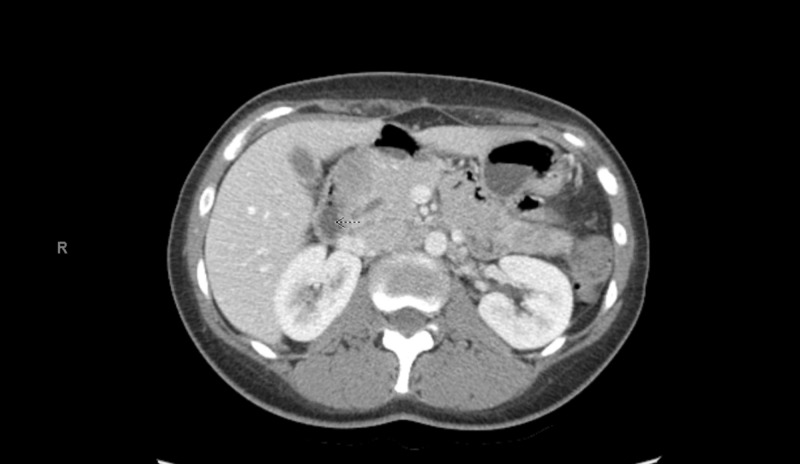
Ill-defined low-density mass lesion within the pancreas, representing a rare feature of sarcoidosis.

Possible interstitial lung disease associated with sarcoidosis, and there were several ill-defined low-density mass lesions within the pancreas (Figure 3). It was thought that it represents a rare feature of sarcoidosis. 

The patient was started on 1 mg/kg of oral prednisone with improvement in her symptoms, including her generalized fatigue, abdominal pain, and her blurred vision and eyes pain. The prednisone was tapered to 10 mg daily and follow-up magnetic resonance imaging after a few months of treatment showed resolution of the pancreatic lesions. The patient was started on methotrexate thereafter with improvement of her symptoms. 

## Discussion

Sarcoidosis is one of the granulomatous diseases that most commonly affects the lungs, but can also affect any other organ of the body. The skin, eyes, joints, heart, central nervous system, and liver are not uncommon organs to be involved [3]. Gastrointestinal tract involvement is most likely to be seen in the liver; however, gastric and intestinal involvement have been documented, clinically mimicking inflammatory bowel disease [4].

Involvement of the pancreas is extremely rare despite being reported in literature [5,6]. It has been noted that 1%-5% of patients with systemic sarcoidosis have pancreatic involvement upon postmortem studies [1]. Due to the rare involvement of the pancreas in the disease process, it remains very difficult and challenging to diagnose it; hence, a high level of clinical suspicion is needed as well as supporting laboratory and imaging studies. A high ACE level and lysozyme levels along with imaging findings can direct to diagnosis [7]. Despite that, pancreatic sarcoidosis is still challenging for the radiologist to diagnose; it can be in a form of direct invasion of the organ, obstruction of the pancreatic or the hepatic biliary ducts causing obstructive jaundice picture, lymphadenopathy of the porta hepatis, and it can also mimic pancreatic adenocarcinoma that may require surgical removal in order to reach the definitive diagnosis [8].

The fact that our patient had systemic sarcoidosis involving the lungs, skin, and eyes, the high ACE level, lysozyme level, a biopsy-proven non-caseating granulomatosis, a great response to steroid therapy, and the resolution of the pancreatic lesions on follow-up MRI all support the diagnosis.

## Conclusions

Sarcoidosis of the pancreas remains a rare disease feature. Despite having the laboratory and imaging tests that might show nodular lesions or a pancreatic mass mimicking adenocarcinoma of the pancreas, it remains challenging to identify it. In cases where there is other organ involvement and a biopsy-proven disease, a suspicion for pancreatic sarcoidosis should be raised in symptomatic patients. The disease usually carries good prognosis and response to steroids. Thus, early identification of the disease will help guide the treatment plan, leading to a decrease in disease morbidity and mortality.
